# Supplementary material for: Synthesis of monodisperse InSb colloidal quantum dots by monomer concentration control for short-wave infrared photodetectors
Source: Nat Commun. 2026 Mar 12;17:3871. doi: 10.1038/s41467-026-70367-6 (PMC13125525; doi:10.1038/s41467-026-70367-6)
Supplement: Supplementary file 1 — Supplementary Information [file 41467_2026_70367_MOESM1_ESM.pdf]

Supplementary Information for

Synthesis of monodisperse colloidal InSb quantum dots by monomer concentration control for short-wave infrared photodetectors

Lucheng Peng[1], Miguel Dosil[1], Debranjan Mandal[1], Hao Wu[1], Aditya Malla[1], Gerasimos Konstantatos[1,2*]

[1]ICFO-Insitut de Ciencies Fotoniques, The Barcelona Institute of Science and Technology, Castelldefels, 08860 Barcelona, Spain.

[2]ICREA-Institució Catalana de Recerca i Estudiats Avançats, Lluis Companys 23, 08010 Barcelona, Spain.

*e-mail: gerasimos.konstantatos@icfo.eu

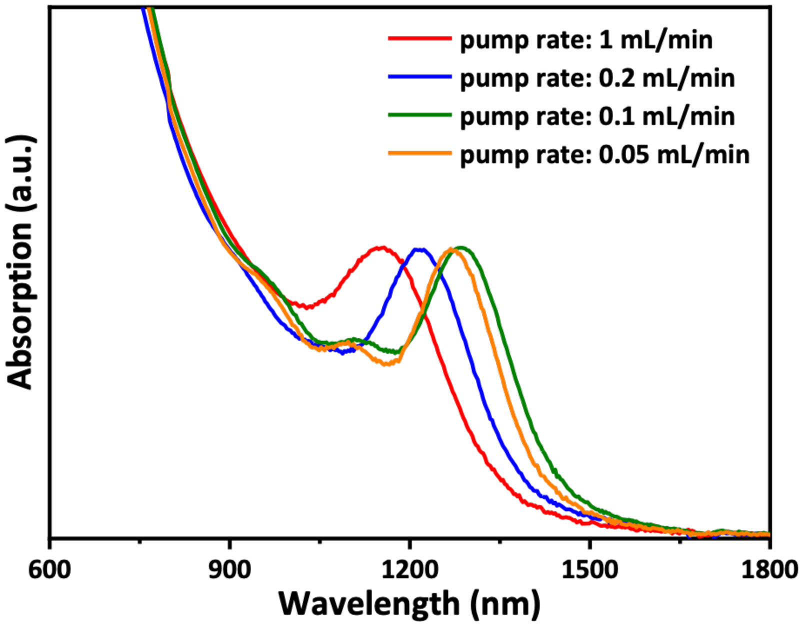

Supplementary Figure 1. Absorption spectra of InSb CQDs with different pump rate for growth at 240 °C. The pumping rate for growth is varied from 1 mL/min to 0.2 mL/min to 0.1 mL/min to 0.05 mL/min, respectively. It shows that the fastest addition rate the QD size distribution significantly broadens, evident from the band-tail absorption and peak-to-valley ratio of the first excitonic peaks. Notably, the faster the pumping rate, the more the absorption spectrum blue-shifts. Considering that the total amount of precursor is fixed, this implies that a faster injection rate induces continuous nucleation, thereby consuming the precursors available for growth and resulting in smaller QD size.

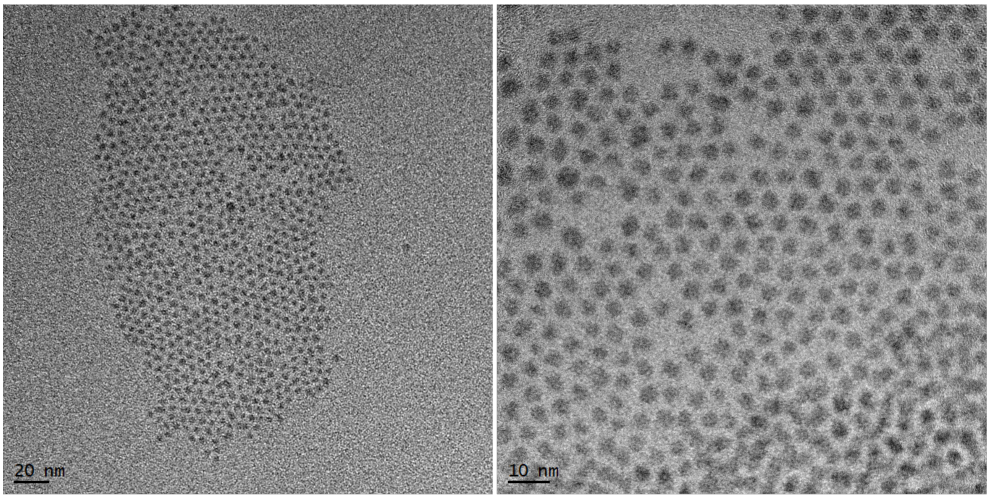

Supplementary Figure 2. The different resolution TEM images of InSb CQDs obtained at 225 °C with excitonic absorption peak at 960 nm.

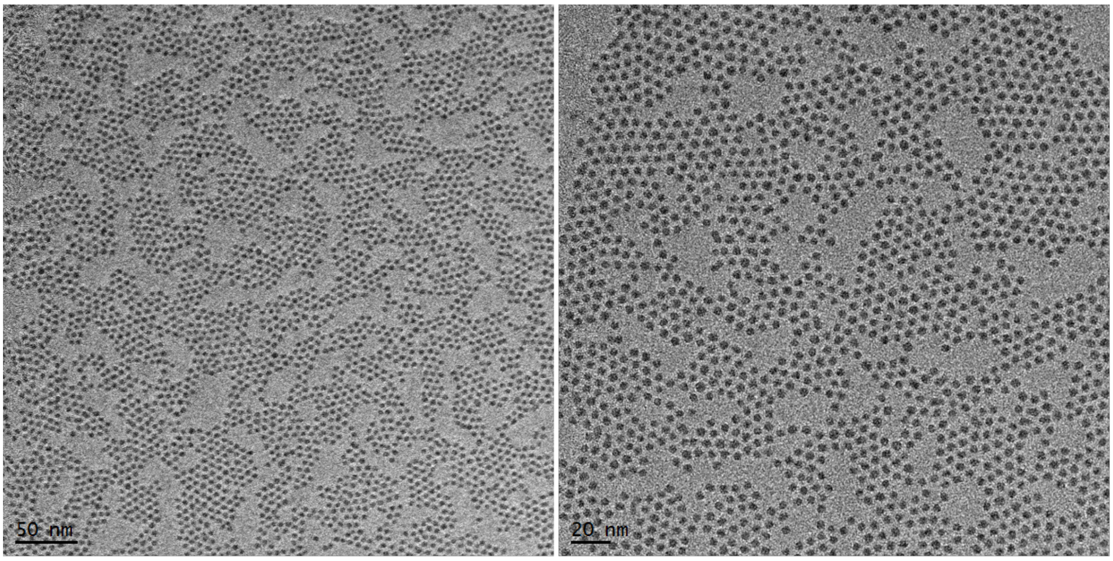

Supplementary Figure 3. The different resolution TEM images of InSb CQDs obtained at 230 ^{o}C with excitonic absorption peak at 1070 nm.

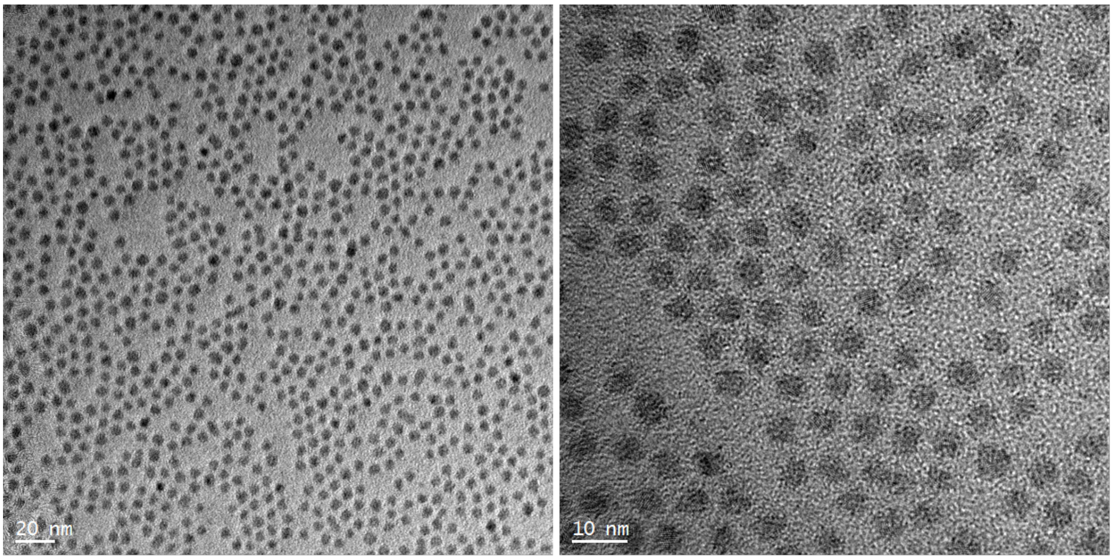

Supplementary Figure 4. The different resolution TEM images of InSb CQDs obtained at 240 ^{o}C with excitonic absorption peak at 1260 nm.

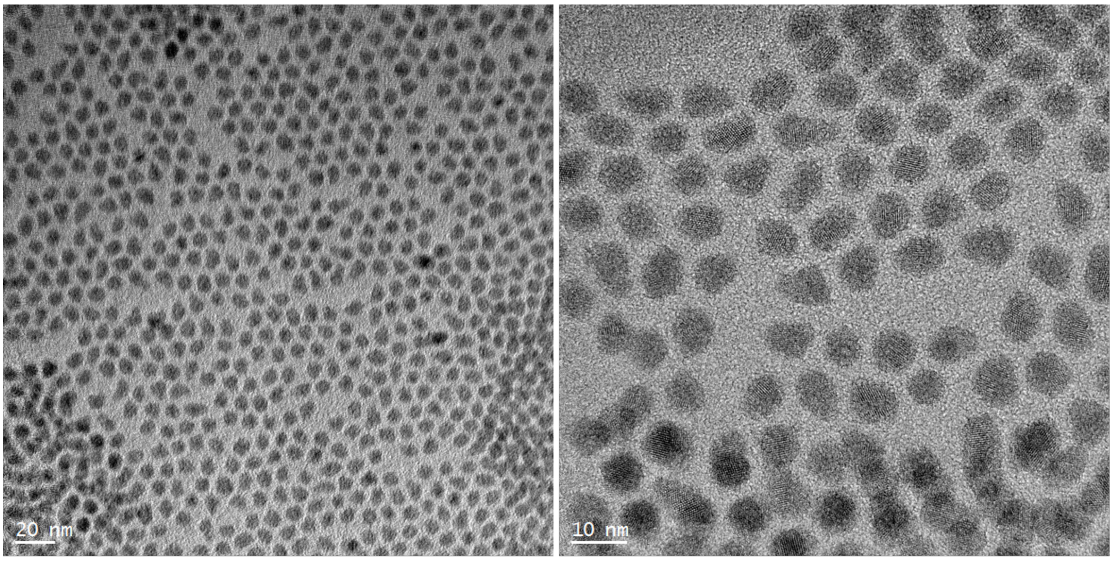

Supplementary Figure 5. The different resolution TEM images of InSb CQDs obtained at 250 ^{o}C with excitonic absorption peak at 1620 nm.

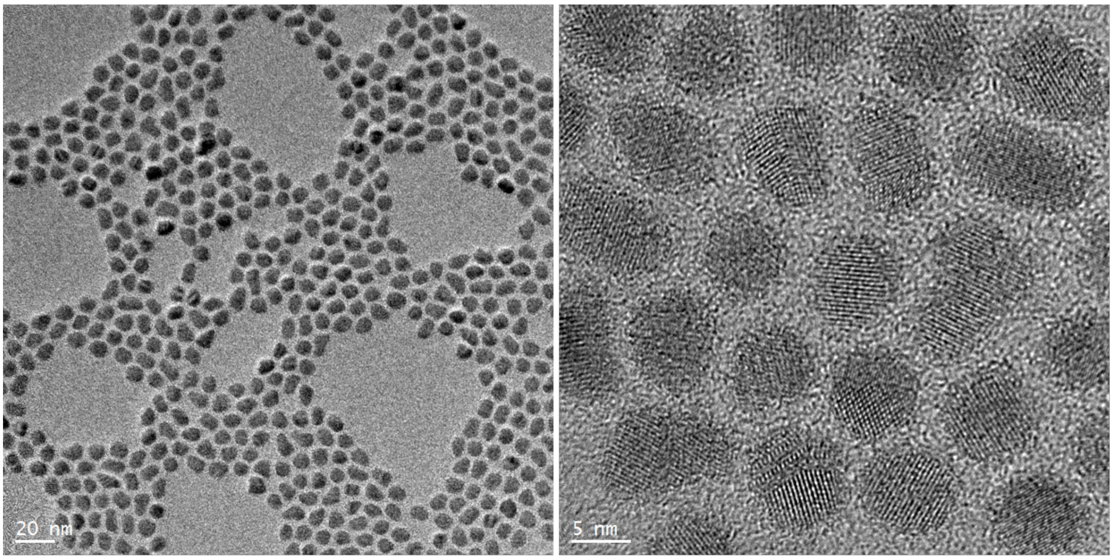

Supplementary Figure 6. The different resolution TEM images of InSb CQDs obtained at 260 ^{o}C with excitonic absorption peak at 1780 nm.

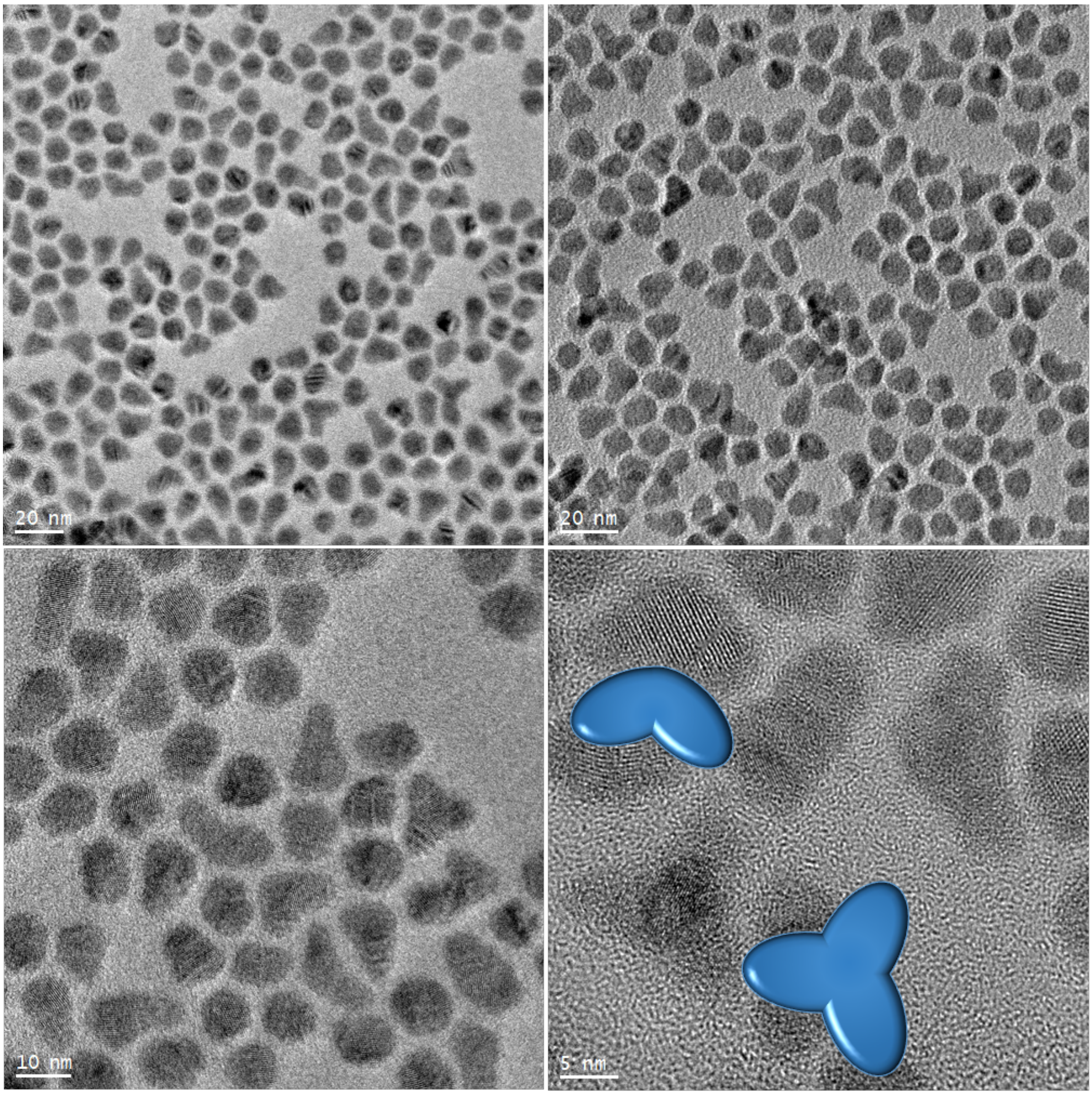

Supplementary Figure 7. The different resolution TEM images of InSb CQDs obtained at 270 °C with excitonic absorption peak at 2250 nm. The quasi-tetrapod shape is observed in TEM images which is likely caused by the partial fusion of QDs at high temperature. Insets in the TEM images show the similar cartoon shape of the dimer and quasi-tetrapod.

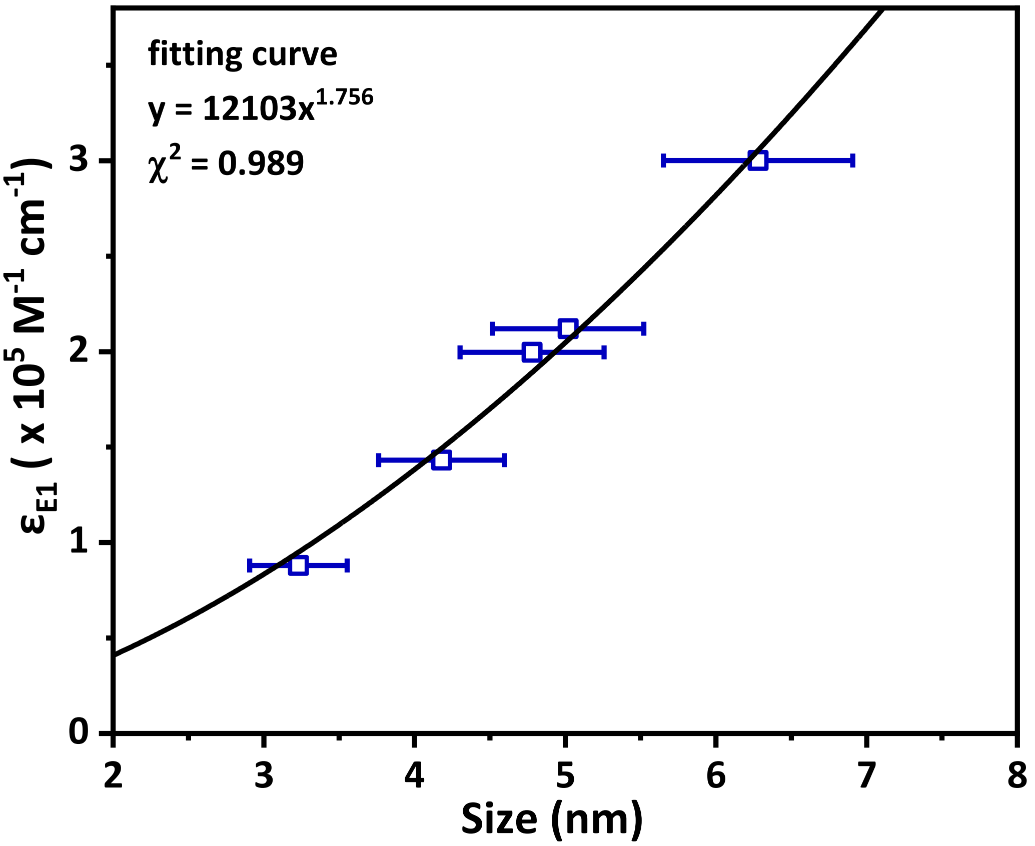

Supplementary Figure 8. Size-dependent molar absorption coefficients (ε_{E1}) of InSb CQDs at the first exciton transition energy. The molar absorption coefficients were determined by digesting the InSb CQDs for inductively coupled plasma (ICP) measurements and calculated according to the Beer-Lambert law. The error bar indicates the size distribution of InSb CQDs.

wavelength (nm)	1019	1280	1618
Size (nm)	2.95	4.29	6.21
absorbance value (A)	0.381	0.245	0.22
ε_{E1} (M^{-1} cm^{-1})	80891	156130	298924
C_{QDs} (µM)	47.1	15.7	7.36

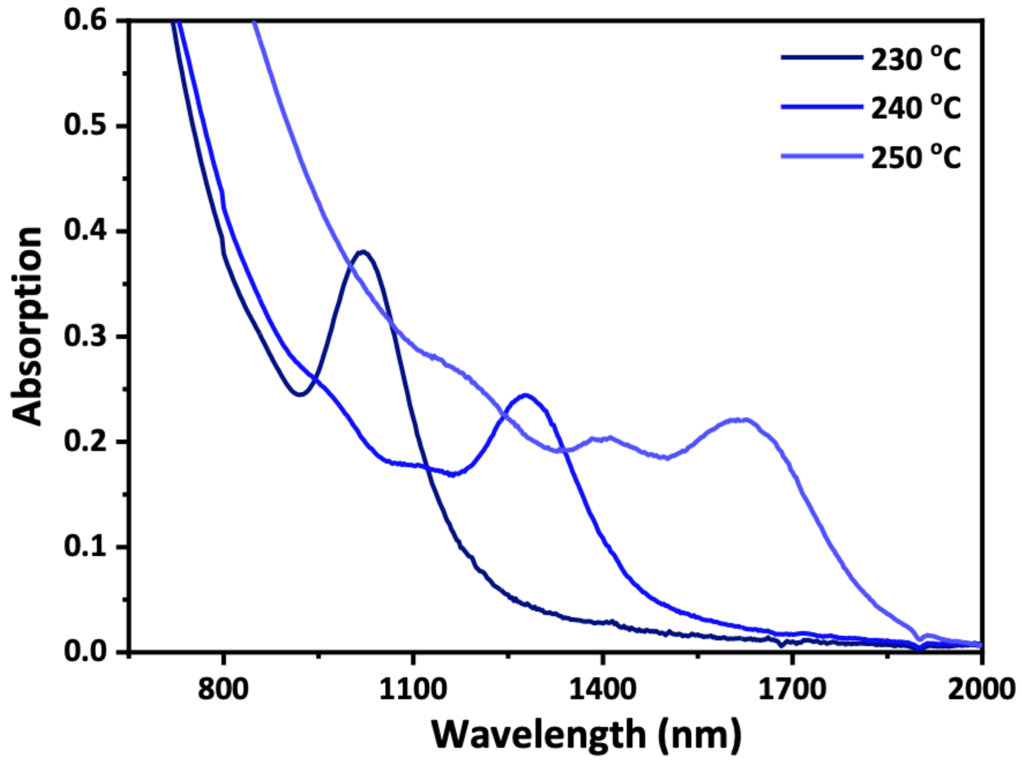

Supplementary Figure 9. The absorption spectra of InSb CQDs (before size-selection) synthesized at 230 °C, 240 °C, and 250 °C using the same precursor amount. Combining the molar absorption coefficients (ε_{E1}) obtained in Figure S8 with the Beer-Lambert law, the concentration of InSb CQDs is calculated. As shown in the table (top), the concentration of InSb CQDs decreased from 47.1 µM to 7.36 µM with the increasing of temperature, indicating fewer nuclei formed, which is consistent with our conclusion.

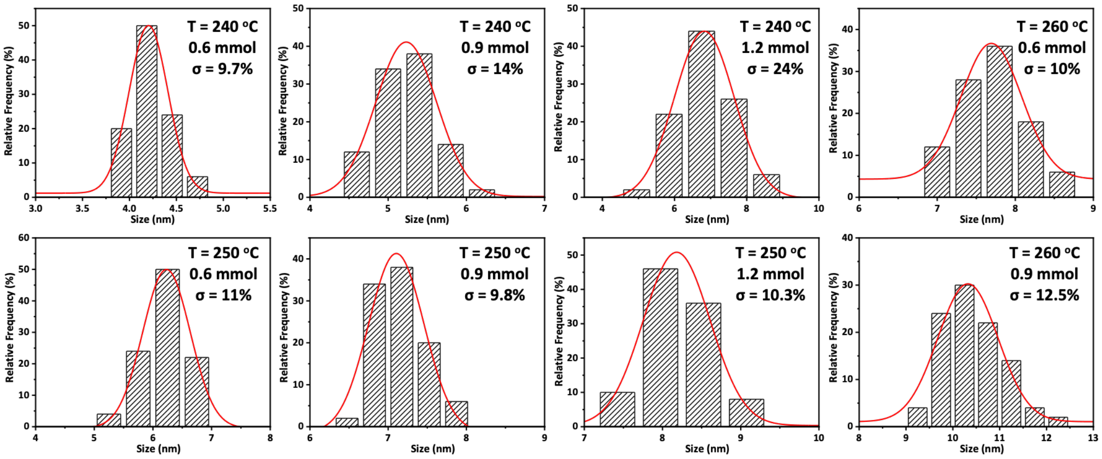

Supplementary Figure 10. Size distribution histograms of the InSb CQDs corresponding to TEM images in Figure 3. The high quality InSb CQDs obtained by the new method shows small size polydispersity around 10%, the broad size distribution under high precursor concentration condition is due to the fusion of QDs.

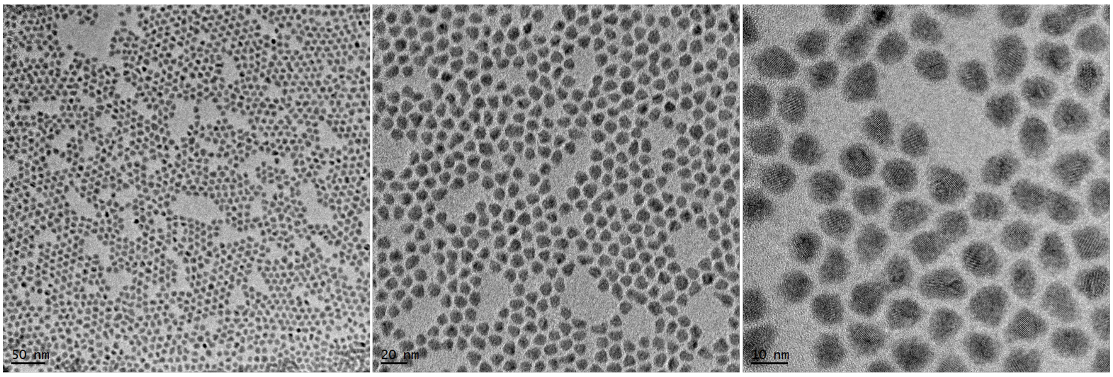

Supplementary Figure 11. The different resolution TEM images of InSb CQDs obtained at 250 °C with excitonic absorption peak at 1900 nm.

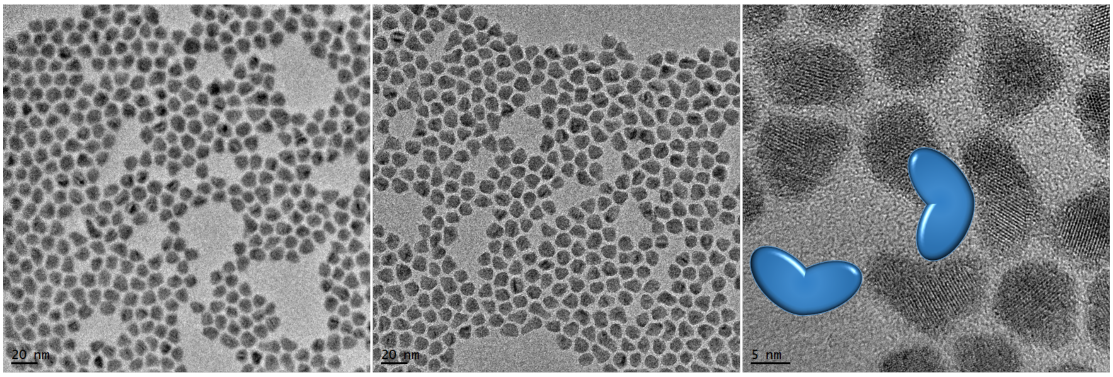

Supplementary Figure 12. The different resolution TEM images of InSb CQDs obtained at 260 °C with excitonic absorption peak at 2300 nm. Insets in the TEM images show the similar cartoon shape of dimer due to the fusion of CQDs.

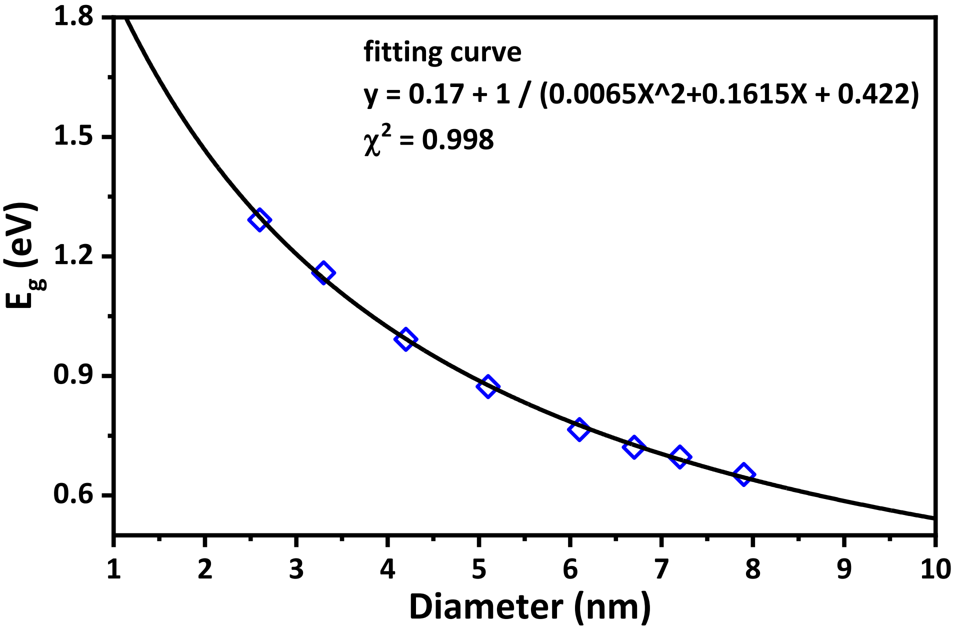

Supplementary Figure 13. Size dependence of the band gap (E_g) of InSb CQDs obtained by MCCA and the fitting with tight-binding calculations by plotting the E_g (*d*) as a function of $1/d^2$.

wavelength (nm)	960	1030	1160	1260	1370	1470	1620	1770
E_g (eV)	1.3	1.2	1.07	0.98	0.90	0.84	0.76	0.7
Size (nm)	2.61	3	3.66	4.18	4.77	5.34	6.22	7.15
$\Delta E_{LH\text{-}HH}$ splitting (meV)	/	/	169	157	137	117	110	96
PL FWHM (meV)	122	115	110	94	96	92	/	/
PL stokes shift (meV)	92	68	56	38	28	18	/	/

Supplementary Table 1. The size-dependent (E_g) light-heavy hole splitting values, PL FWHM, and Stokes shifts.

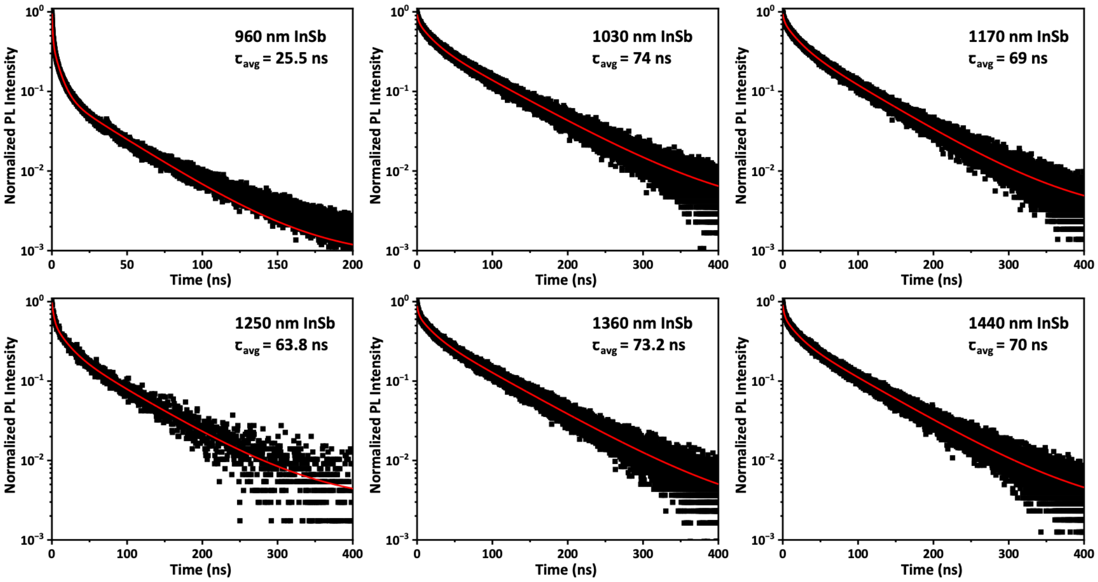

Supplementary Figure 14. The photoluminescence intensity decay spectra of representative InSb CQDs with band gap ranging from 1.21 eV to 0.843 eV. The PL decay trace is fitted with the fast component, presumably corresponding to some nonradiative recombination channel, followed by a slow nearly monoexponentially component with average 70 ns time that likely corresponded to the radiative lifetime.

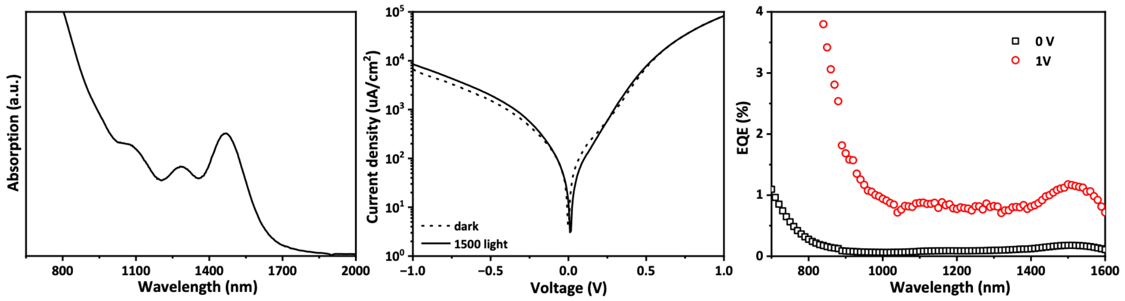

Supplementary Figure 15. 1465 nm InSb CQDs photodetector characterizations. Current density-voltage (*J-V*) curves of InSb CQDs photodetector dark and under 1500 nm illumination with power densities of 47 mW cm^{-2}, and the corresponding EQE spectra under 0 V bias and -1 V bias.

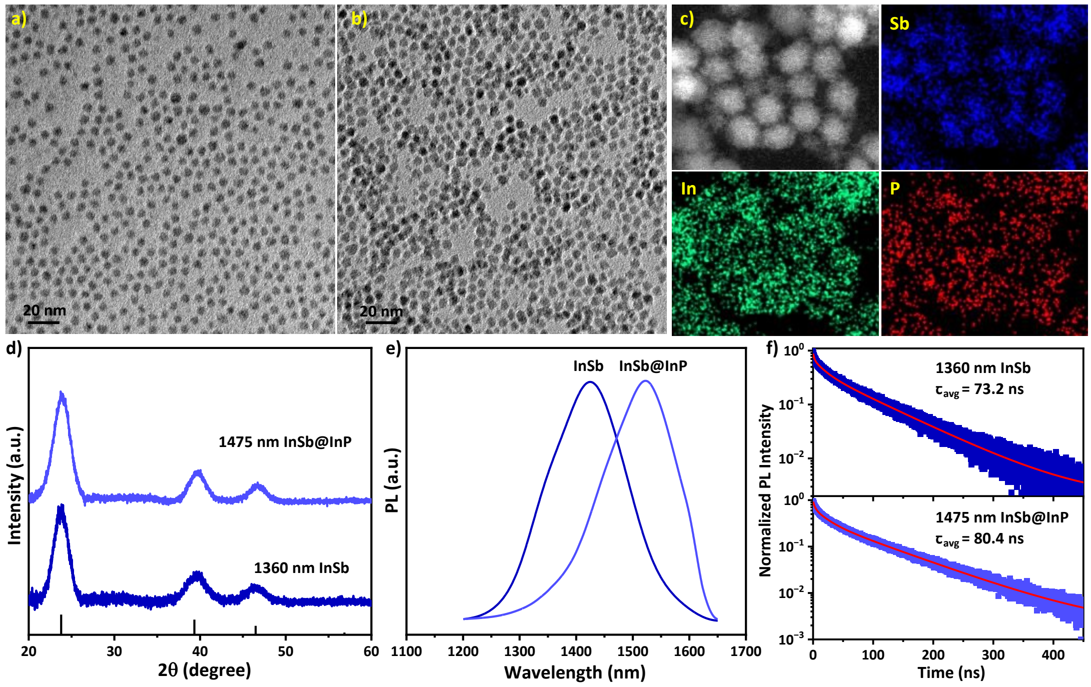

Supplementary Figure 16. Characterizations of 1360 nm InSb CQDs before and after InP shell growth. TEM images of InSb CQDs (a) and InSb@InP core-shell CQDs (b). (c) STEM image of InSb@InP core-shell CQDs and the corresponding EDX-mapping that shows the atomic ratio of In : Sb : P is 56 : 30 : 14. (d) XRD patterns of InSb CQDs before and after InP shell growth. (e) PL spectra of InSb CQDs before and after InP shell growth, and it shows the PLQY improves from 2% to 6.8% according to the integrating sphere calculation method. (f) The PL lifetime of InSb CQDs before and after InP shell growth.

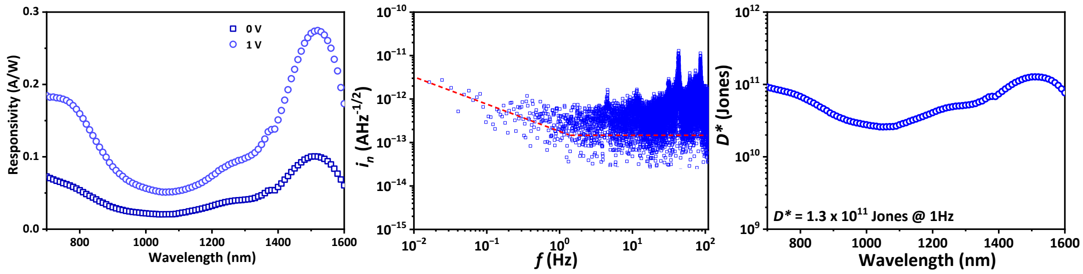

Supplementary Figure 17. Device performance characterizations of 1475 nm InSb/InP core-shell CQDs based photodetector. The responsivity spectra (left) of the device with the bias of 0 V and 1 V (reverse bias). Frequency dependent noise spectral density of the device at zero bias (middle), which is measured by transient-current fast Fourier-Transform (FFT) method. The obtained noise spectrum shows a 1/f noise dominating at low frequency and reaches a flat noise floor of ~ 1.4×10^{-13} A $Hz^{-0.5}$ at a frequency of 1 Hz. Specific detectivity spectrum ($D^* \sim 1.3 \times 10^{11}$ Jones) of the device (right) at 1 Hz and 0V bias conditions.

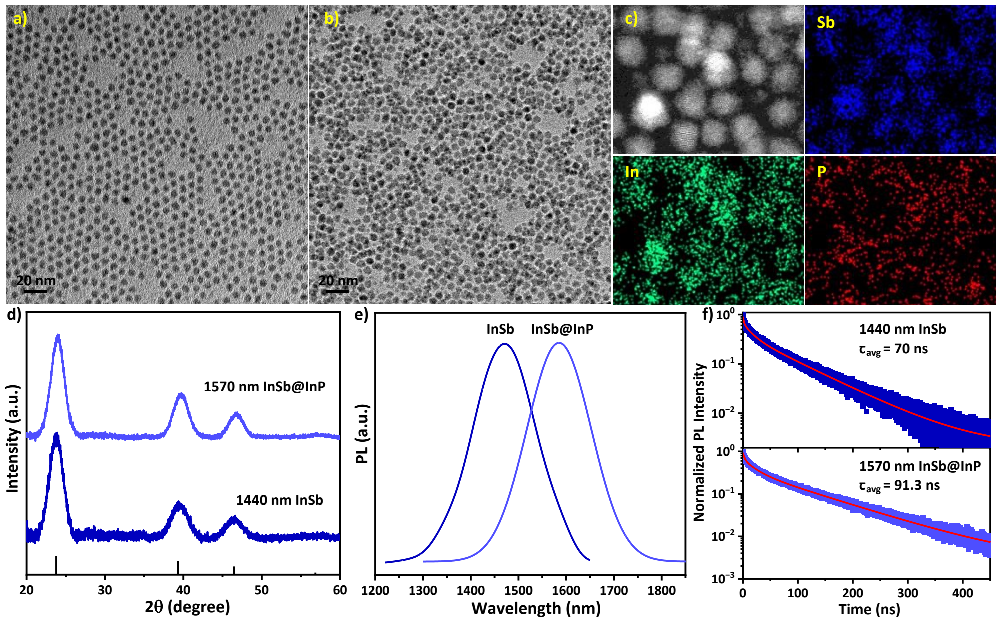

Supplementary Figure 18. Characterizations of 1440 nm InSb CQDs before and after InP shell growth. TEM images of InSb CQDs (a) and InSb@InP core-shell CQDs (b). (c) STEM image of InSb@InP core-shell CQDs and the corresponding EDX-mapping that shows the atomic ratio of In : Sb : P is 54 : 30 : 16. (d) XRD patterns of InSb CQDs before and after InP shell growth. (e) PL spectra of InSb CQDs before and after InP shell growth, and it shows the PLQY improves from 1.5% to 5.2% after shelling. (f) The PL lifetime of InSb CQDs before and after InP shell growth.

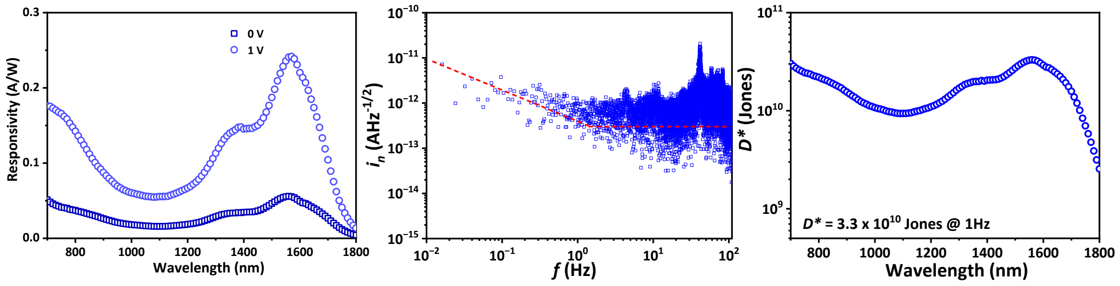

Supplementary Figure 19. Device performance characterizations of 1570 nm InSb/InP core-shell CQDs based photodetector. The responsivity spectra (left) of the device with the bias of 0 V and 1 V (reverse bias). Frequency dependent noise spectral density of the device at zero bias (middle), which is measured by transient-current fast Fourier-Transform (FFT) method. The obtained noise spectrum shows a 1/f noise dominating at low frequency and reaches a flat noise floor of ~ 3 × 10^{-13} A Hz$^{-0.5}$ at a frequency of 1 Hz. Specific detectivity spectrum (D* ~ 3.3 × 10^{10} Jones) of the device (right) at 1 Hz and 0V bias conditions.

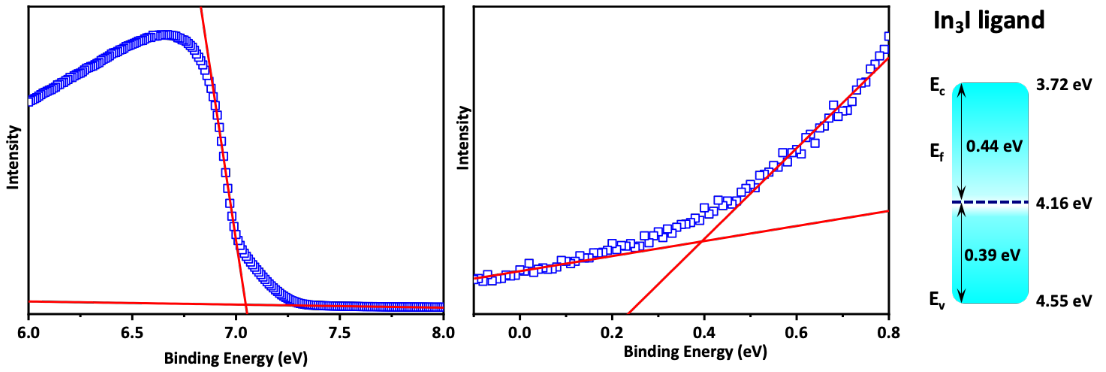

Supplementary Figure 20. The energy level of 1475 nm InSb/InP core-shell CQDs films analyzed by the UPS spectra after InI_3 ligand exchange.

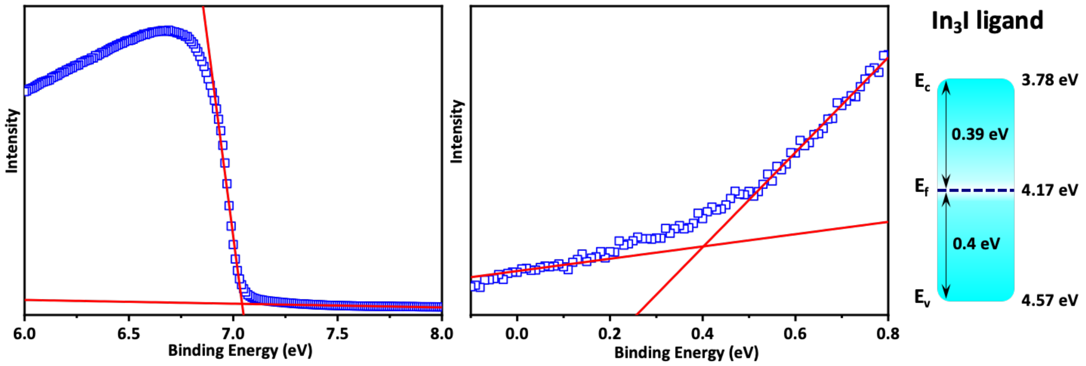

Supplementary Figure 21. The energy level of 1570 nm InSb/InP core-shell CQDs films analyzed by the UPS spectra after InI_3 ligand exchange.

wavelength (nm)	1050	1148	1260	1364	1464
Size (nm)	3.1	3.6	4.18	4.76	5.32
amount of precursor for synthesis (mmol)	0.6	0.6	0.6	0.6	0.6
obtained InSb CQDs after purification (mg)	112	104	100	106	89
reaction yield (%)	78.8%	73.2%	70.4%	74.6%	63%

Supplementary Table 2. The reaction yields of different InSb CQDs sizes. The reaction yield is calculated by dividing obtained InSb CQDs after purification (mg) by 0.6 mmol InSb precursors.
